# Loss of BAP1 Is Associated with Upregulation of the NFkB Pathway and Increased HLA Class I Expression in Uveal Melanoma

**DOI:** 10.3390/cancers11081102

**Published:** 2019-08-02

**Authors:** Zahra Souri, Annemijn P. A. Wierenga, Christiaan van Weeghel, Pieter A. van der Velden, Wilma G. M. Kroes, Gregorius P. M. Luyten, Sjoerd H. van der Burg, Aart G. Jochemsen, Martine J. Jager

**Affiliations:** 1 Department of Ophthalmology, Leiden University Medical Center, Albinusdreef 2, 2333 ZA Leiden, The Netherlands; 2 Department of Clinical Genetics, Leiden University Medical Center, 2333 ZA Leiden, The Netherlands; 3 Department of Clinical Oncology, Leiden University Medical Center, 2333 ZA Leiden, The Netherlands; 4 Department of Cell and Chemical Biology, Leiden University Medical Center, 2333 ZA Leiden, The Netherlands

**Keywords:** uveal melanoma, inflammation, HLA Class I, NFkB pathway, BAP1, oncology

## Abstract

One of the characteristics of prognostically infaust uveal melanoma (UM) is an inflammatory phenotype, which is characterized by high numbers of infiltrating T cells and macrophages, and a high HLA Class I expression. We wondered how this inflammation is regulated, and considered that one of the most important regulators of inflammation, the NFkB pathway, might play a role. We analyzed 64 UM samples for expression of HLA Class I, its regulators, and of members of the NFkB transcription family, using an Illumina HT12V4 array. HLA Class I expression and infiltrating immune cells were also determined by immunohistochemical staining. Information was obtained regarding chromosome status by Affymetrix Nsp array. Our analysis shows that expression of NFkB1, NFkB2 and RELB positively correlates with the level of HLA Class I expression and the number of infiltrating T cells and macrophages, while SPP1 and PPARγ are negatively correlated. Increased levels of NFkB1 and NFkB2 and decreased levels of SPP1 and PPARγ are seen in Monosomy 3/BAP1-negative tumors. This is also the case in non-inflammatory UM, indicating that our observation not only involves infiltrating leukocytes but the tumor cells themselves. We report that the NFkB pathway is associated with inflammation and HLA Class I expression in UM, and is upregulated when BAP1 expression is lost.

## 1. Introduction

Uveal melanoma (UM) is a malignancy that originates from melanocytes in the eye, and 50% of the patients will ultimately develop metastases [1,2]. Immune cell infiltration, secretion of inflammatory cytokines and an increase in HLA expression are frequently observed in UM, leading to an inflamed tumor environment, inside the immune-privileged eye [3]. This inflammatory phenotype is linked to an increased risk for metastasis [4,5,6,7], and has been associated with loss of chromosome 3 [8,9]. It was recently noticed that an extra copy of chromosome 8q in the tumor is associated with an influx of macrophages, while loss of chromosome 3 (Monosomy 3, M3) correlates with an increased influx of T cells [10]. M3 occurs in almost 50% of patients and is associated with a bad prognosis [11,12,13]. Almost all cases with M3 also show gain of chromosome 8q, but this may even occur in tumors with two chromosomes 3 (Disomy 3, D3); it is considered a bad prognostic sign [14,15,16]. Another alteration is a gain of 6p, which occurs in 25–40% of cases, and is a sign of good prognosis [17,18].

Besides chromosomal changes, important genetic driver mutations have been identified: these include activating mutations in *GNAQ* and *GNA11* [19,20], which are thought to lead to the transformation of melanocytic cells by upregulation of YAP1 [21]. These mutations are already seen in choroidal nevi, where YAP upregulation is also present [22]. Loss of expression of a key tumor suppressor gene, *BAP1*, encoding a deubiquitinating protein and located on chromosome 3, together with the loss of the other chromosome 3, is strongly correlated with the development of metastases [23]. Major signalling pathways such as the Rb, PI3K/Akt, MAPK and NFkB pathways are frequently disregulated in prognostically bad UM [24,25,26]. This disregulation will lead to inhibition of apoptosis and stimulation of cell proliferation, while the cells will become more invasive and tend to migrate to other organs.

Inflammation is considered one of the hallmarks of cancer [27], but its regulation is not always clear. Although in UM a relation is seen between M3/loss of BAP1 expression and the presence of an inflammatory phenotype, little is known about the pathways which regulate this inflammation. One of the major regulators of inflammation in cancer is the NFkB-signalling pathway [28]. Members of the NFkB transcription family include RELA (p65), RELB, c-REL, NFkB1 (p105/p50), and NFkB2 (p100/p52), which are regulated by proteins such as IKK and NEMO (IkBkG). The canonical pathway, in which NFkB1 is the main player, is involved in many different aspects of the immune system while the non-canonical pathway, in which NFkB2 is involved, regulates specific functions of the adaptive immune system.

During tumorigenesis, the NFkB pathway is often upregulated and leads to uncontrolled proliferation, apoptosis resistance, angiogenesis, inhibition of senescence, invasion, metastasis and resistance to different treatments [28]. P53 and PTEN proteins can function as negative regulators of NFkB signalling and mutations in these genes can affect the pathway’s activity. Other oncogenic mutations, such as amplifications and point mutations in *RELA* and other *NFkB* signalling genes, have been identified in several lymphoid malignancies, and give rise to inflammation [29]. Stromal M2 macrophages produce various cytokines and play a major role in NFkB upregulation. In cutaneous melanoma, the NFkB pathway is known as a molecular switch which is turned on during tumor progression [30,31].

Both the canonical and non-canonical NFkB pathway have been shown to be active in primary and metastatic UM and are related to progression of the disease [32,33,34], while inhibition of NFkB has been shown to reduce cell proliferation. As far as we know, the relation between NFkB signalling, HLA Class I expression and genetic tumor progression in UM has not been reported. As we observed in our previous studies that genetic aberrations in UM are related to progression of the inflammatory environment, we set out to investigate whether NFkB plays a role in the expression of HLA Class I and whether the expression of NFkB itself is related to the tumor’s chromosome 3/BAP1 status.

## 2. Results

### 2.1. HLA Expression is Related to NFkB Pathway Molecules

As an increased HLA expression is one of the hallmarks of the inflammatory phenotype in UM, we wondered whether the NFkB pathway plays a role in the expression of HLA Class I (A, B) molecules. We investigated this by analysis of Illumina mRNA expression data. As we had several probes for the HLA Class I markers, we compared these probes with previously acquired immunohistochemistry data (Supplementary Table S1) [8,35,36]. Three of four mRNA probes for gene expression of HLA-A correlated with the immunohistochemical data on HLA-A. The probe for HLA-B correlated with HC10 staining (*p* = 0.001). Using the three best HLA-A probes and the HLA-B probe, we determined the relation between the NFKB pathway and HLA expression in UM (Table 1).

Significant positive correlations were seen between most of the NFkB markers and HLA Class I: NFkB1, NFkB2-pr1 (probe 1) and RELB had a significant positive correlation with HLA-A and HLA-B. SPP1-pr2 (probe 2) showed a negative correlation with HLA-B, while PPARγ-pr1 showed a negative correlation with HLA-A.

### 2.2. NFkB Pathway is Associated with HLA Class I Regulatory Factors and the Antigen-Loading Machinery

In order to further investigate the relationship between HLA Class I expression and the NFkB pathway, we investigated a putative relation between the NFkB-signalling pathway molecules and HLA Class I regulatory factors (Table 2). CIITA and NLRC5 are both known as regulators of HLA Class I expression. IRF1, IRF2 and IRF8 are all transcription factors which specifically bind to the interferon response element and are known to play a role in the induction of HLA Class I expression in many tissues [35]. TAP1, TAP2, Tapasin and Calreticulin all belong to the antigen-loading machinery and play a role in the assembly of HLA Class I molecules on the cell surface [37].

NFkB1 showed a correlation with IRF1 and TAP1, while NFkB2 had positive correlations with TAP1 and Calreticulin. RELA did not show any significant correlations while RELB showed positive correlations with IRF1, IRF8, TAP1 and TAP2. Both probes of SPP1 showed negative correlations with IRF1 and TAP1. Both probes of PPARγ showed negative correlations with IRF1, IRF8 and TAP1.

### 2.3. Expression of NFkB Pathway Molecules is Related to the Presence of Infiltrating Leukocytes

In order to investigate whether the NFkB pathway is involved in the regulation of leukocyte influx, we determined whether any correlation could be found between expression of members of the NFkB pathway and markers of infiltrate (determined by immunohistochemistry and mRNA analysis). Since several probes for T cell and macrophage markers were available, we looked at data from a comparison between previous immunofluorescence results and gene expression data and selected the probes with the highest correlation [9,10,38]. We decided to use the following markers to determine the presence of infiltrating leukocytes: CD3E and CD3D1 (part of the T-cell receptor-CD3 complex as a marker for T cells), CD4 as a marker for T-helper cells and macrophages, CD8A as a marker for cytotoxic T cells, and CD68 and CD163 as markers for monocytes and macrophages (Table 3).

Expression of NFkB1 was positively correlated with the presence of CD3 and CD8 T cells (CD3E *p* = 0.01, CD8A *p* = 0.03). Similarly, both probes for NFkB2 had a positive correlation with CD3E and CD68. RELA did not show any significant correlation, while expression of RELB was significantly correlated to CD3E and CD3D1. Both SPP1 probes showed a negative correlation with CD68, while expression of PPARγ (both probes) showed a negative correlation with CD4 and CD163. Expression of IkBkG showed a positive correlation with CD163.

### 2.4. Confirmation of Associations Found by Heatmap and Survival

#### 2.4.1. Distribution of NFkB Pathway and HLA Class I Gene Expression in Primary UM

**Figure 1:**
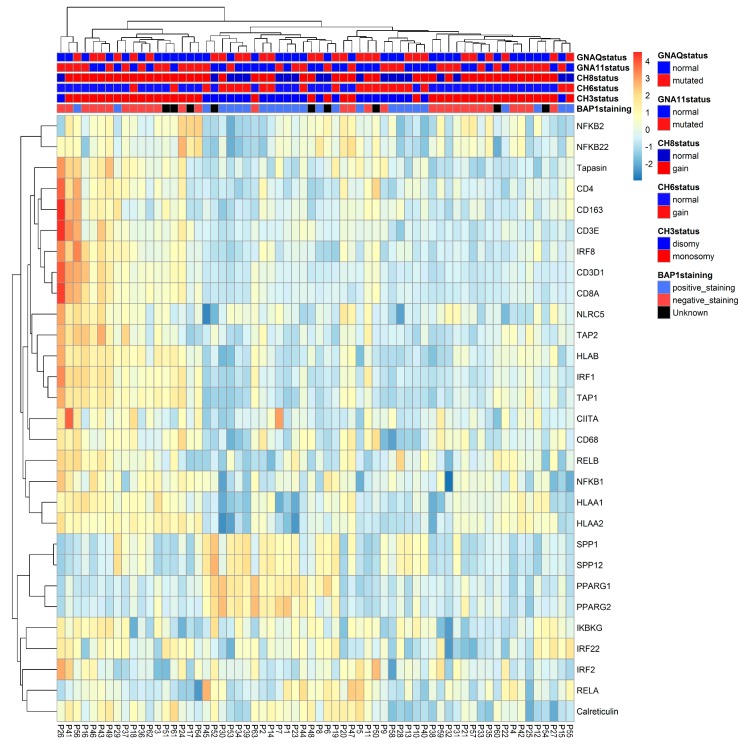
Heat map of 64 UM, highlighting mRNA expression levels of inflammatory markers. The presence of BAP1 staining, the status of chromosome 3, 6p and 8q, and the presence of GNAQ and GNA11 mutations are also shown. Clusters were created by unsupervised clustering of the tumors.

We put all different analyses together in a heat map, which displays the data of 64 UM, highlighting mRNA expression levels of relevant immune markers and tumor characteristics. According to the heatmap (Figure 1), most D3/BAP1-positive tumors (indicated as blue in the legend) had low expression of HLA-A, HLA-B, NFkB1, NFkB2 (indicated as blue in the expression array) and high levels of SPP1 and PPARγ (indicated as yellow-orange) while most M3/BAP1-negative tumors (indicated as red in the legend) had a high expression of HLA-A, HLA-B, NFkB1, NFkB2 (indicated as yellow-orange) and low levels of SPP1 and PPARγ (indicated as blue).

#### 2.4.2. Associations between Survival in UM and Chromosome 3, BAP1 and Inflammatory Factors

**Figure 2:**
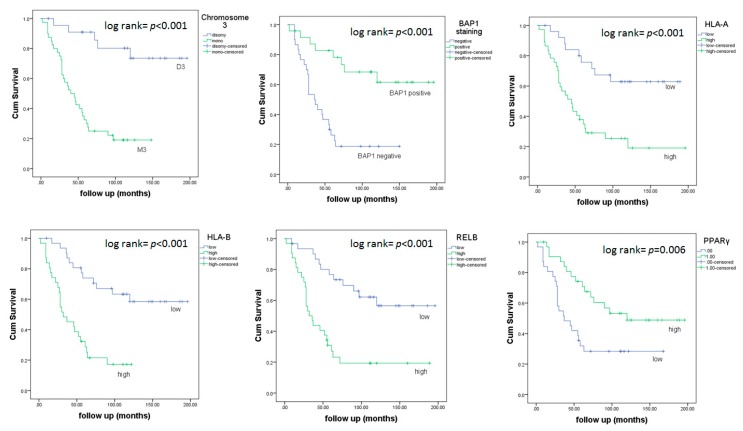
Kaplan–Meier survival curves based on chromosome 3 status, BAP1 staining and mRNA expression of HLA-A, HLA-B, RELB and PPARγ. A log-rank test was used for the significance analysis.

Next, we investigated whether any differences could be found between survival with regard to the level of expression of HLA Class I and NFkB pathway molecules. Different levels of expression in HLA-A, HLAB, RELB and PPARγ significantly differed in survival (Figure 2). Tumors with a low expression of HLA-A, HLA-B and RELB led to a better overall survival (*p <* 0.001, *p <* 0.001, *p <* 0.001), while tumors with a high level of PPARγ correlated with better survival (*p* = 0.006).We also checked survival in relation to chromosome 3 status and BAP1 staining. Patients with M3 tumors showed a much shorter survival compared to patients with D3 tumors (*p <* 0.001), and those with tumors that lacked BAP1 staining had a shorter survival compared to those with BAP1-positive tumors (*p <* 0.001).

### 2.5. NFkB Pathway and Chromosome 3/BAP1 Status

**Figure 3:**
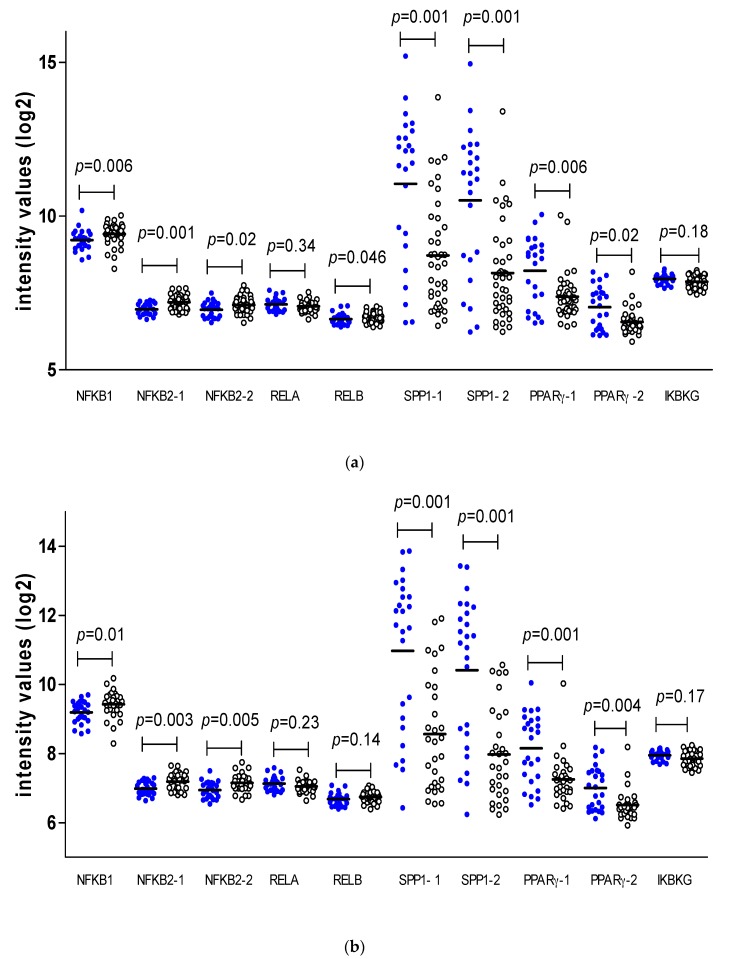
Comparison of expression of factors involved in NFkB signalling pathways in (**a**) D3 (● blue) (*n* = 24) versus M3 (o white) (*n* = 40), and (**b**) BAP1-positive (● blue) (*n* = 25) versus BAP1-negative (o white) (*n* = 30) tumors. Mann–Whitney U test, *p* ≤ 0.05 is considered significant. Thick bars indicate the means.

As M3 and loss of BAP1 expression are well known risk factors for the development of metastasis in UM, we determined whether these genetic aberrations might be associated with expression of the NFkB-related molecules directly and, therefore, with the generation of the inflammatory environment. First, we analysed the gene expression levels of the NFkB-related markers versus chromosome 3 status in our cohort of 64 tumors (Figure 3a). M3 was associated with an increase in NFkB1, NFkB2, and RELB and a decrease in SPP1 (both probes) and PPARγ (both probes).

Next, we analysed a group of 55 tumors with information on BAP1 staining (Figure 3b). Levels of NFkB1 and NFkB2 (both probes) were increased in tumors with BAP1 loss, while SPP1 (both probes) and PPARγ (both probes) were decreased in these tumors.

### 2.6. NFkB Upregulation is Present in Tumor Cells

However, the positive correlations may be (partially) due to the expression of NFkB in infiltrating lymphocytes and macrophages. In order to determine whether the association between loss of BAP1 and changes in the NFkB pathway occurs in the tumor cells themselves, we excluded the 33% of tumors with the highest CD8 score, leaving a total of 43 tumors. Even with the decreased number of cases, one probe for NFkB2 was still positively correlated with two HLA-A probes and with HLA-B, while SPP1 and PPARγ probes remained significantly negatively correlated to some or all of the HLA probes (Table 4).

Most of the associations also remained significant when we performed the same comparison after exclusion of one-third of the tumors with the highest number of macrophages (CD68) (Supplementary Table S2).

We subsequently compared the levels of expression of NFkB pathway molecules in BAP1-positive and -negative tumors, using the whole panel, as well as after exclusion of one-third of the tumors with the highest CD8 scores. BAP1-negative tumors still had higher levels of NFkB2 and IkBkG, while the negative correlations with SPP1 and PPARγ remained (Table 5).

## 3. Discussion

We hypothesized that the inflammatory phenotype with an increased HLA Class I expression in UM is linked to the activation of the NFkB pathway. Therefore, we explored the expression of some of the most important components of the NFkB signalling pathway and investigated their association with HLA-A and HLA-B expression and with different types of infiltrating leukocytes. In accordance with previous studies, we noticed an association between upregulation of most of the factors involved in the NFkB pathway with inflammatory factors [33,34]. We found that NFkB1, NFkB2 and RELB expression were positively correlated with the expression levels of HLA-A and HLA-B, their regulators and their antigen-loading machinery, suggesting that these NFkB pathway molecules may be positive regulators of HLA-A and HLA-B transcription. It has been suggested that NFkB is responsible for the secretion of several proinflammatory cytokines and chemokines and thereby guarantees the attraction, activation, survival and differentiation of immune cells [39] which might lead to the increase of HLA Class I expression in the tumor cells. Singh et al. [34], in a recent study, similarly observed that infiltration increases during progression of UM and that the canonical NFkB pathway is more active in high-risk inflamed UM. They did not report on the chromosome or BAP1 status of the tumors they studied.

We observed that M3 tumors showed higher expression levels of NFkB1, NFkB2, and RELB. Similarly, BAP1-negative tumors showed higher levels of NFkB1 and NFkB2. We previously demonstrated that M3/loss of BAP1 is related to increased leukocytic infiltration [8,9], and now show an association between BAP1 loss and upregulation of the NFkB pathway. As the link between the expression of the NFkB molecules and T cell infiltrate may also be caused by the expression of these molecules in the immune infiltrate itself, we repeated the analyses on the same set but now without one-third of the tumors, which based on the expression of CD8, were highly infiltrated with T cells. We again looked for associations between HLA and NFkB, and the genetic constitution. In this group, we similarly observed that the tumors with M3/loss of BAP1 had an increased NFkB and HLA Class I expression, indicating that the infiltrating leukocytes may enhance expression, but that the genetic basis determines expression in the first place.

We also explored the presence of negative regulators of inflammation in UM: SPP1 and PPAR-γ. The SPP1/osteopontin protein is involved in different aspects of tumor biology such as cell survival and proliferation, invasion and metastasis, and is involved in neural crest development. Kadkol et al. [40] reported that the level of SPP1 expression was increased in the serum of patients with UM metastasis, and that tumors with looping high-risk vasculogenic mimicry patterns had increased expression of osteopontin. Another study [41] also looked at protein expression using a monoclonal antibody and did not observe a relation between expression and survival. In contrast to Kadkol and Simoes, we looked at mRNA and observed that decreased levels of SPP1 mRNA were associated with an unfavorable prognosis in UM. Onken et al. have similarly reported a decreased expression of SPP1 mRNA in high-risk UM [42]. It is of interest to further investigate the mechanism of SPP1 downregulation during UM progression to determine its function.

**Figure 4:**
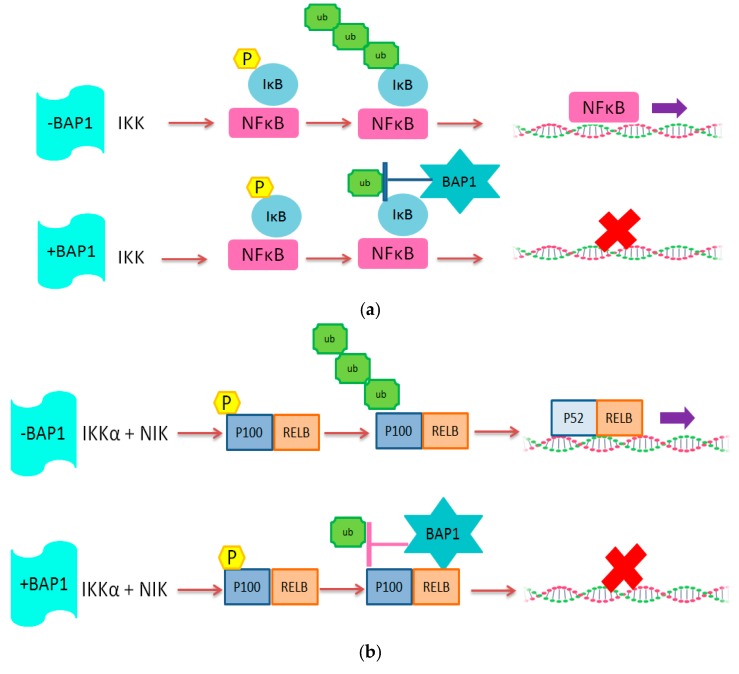
Schematic illustration of the potential role of BAP1 protein in (**a**) Canonical and (**b**) non-Canonical NFkB pathway. We propose that BAP1, as a deubiquitinating enzyme, inhibits NFkB activity.

The upregulation of the NFkB pathway upon loss of one copy of chromosome 3 could possibly be explained by the fact that the *PPAR-*γ gene is located on chromosome 3. The PPAR-γ protein is a negative regulator of the NFkB pathway, so it has been hypothesized that loss of chromosome 3 leads to insufficient inactivation of NFkB by PPAR-γ [43]. In our study, SPP1 and PPAR-γ expression were negatively correlated to T lymphocyte and macrophage markers. As expected, we found a positive correlation between the expression of these two factors with BAP1 expression. We previously observed [35] that a high expression of HLA-A, HLA-B, B2M, TAP1, IRF1 and IRF8 was related to an increase in the presence of T cells. van Essen and colleagues compared the expression levels of these genes in primary tumors with their corresponding xenograft in immunodeficient mice, in which, of course, no human leukocytes are present. They showed that the expression of HLA-A, HLA-B, B2M, TAP1, TAP2, IRF1, IRF8, CIITA and NLRC5 were all down-regulated in xenografts compared to the primary tumors, suggesting that the increased expression depended on the presence of infiltrate. Cytokines potentially involved in the upregulation of these genes were interferon-gamma and CCL2. In the current study, we observed a correlation between the expression of the HLA and HLA-associated genes, the presence of infiltrating immune cells, and the NFkB pathway, but we also see this correlation in tumors without inflammation. From this, we conclude that the genetic basis of the tumor, i.e., Monosomy 3 with BAP1 loss, determines the primary upregulation of the NFkB pathway, which leads to an increase in HLA Class I expression in the tumor cells and production of cytokines and chemokines, which then attracts immune cells, further upregulating expression of HLA Class I. These data indicate that absence of the deubiquitinating function of BAP1 allows the NFkB pathway to be active, potentially by affecting the regulators of NFkB directly (Figure 4).

These findings imply that BAP1 (UCHL2) might have a similar function in UM as observed for UCHL1 in Human papilloma virus (HPV)-infected keratinocytes. UCHL1 and UCHL2 are both ubiquitin hydrolase enzymes which belong to the ubiquitin hydrolase family [44]. HPV-infected keratinocytes produce increased levels of the ubiquitin protease UCHL1, which reduces tumor necrosis factor receptor-associated factor 3 (TRAF3) K63 poly-ubiquitination, resulting in the suppression of the production of interferon and other pro-inflammatory cytokines [45]. Higher UCHL1 expression also resulted in increased degradation of an essential NFkB modulator, IkBkG. In this manner, canonical NFkB signalling was reduced, thereby suppressing a potential anti-viral immune response. HPV-infected keratinocytes with a high UCHL1 expression showed greatly decreased HLA-A and -B levels [46]. The virus uses UCHL1 to escape from the immune system, while in UM, the presence of an inflammatory infiltrate helps to produce blood vessels [9].

Taken together, our data suggest that the main regulator of the NFkB pathway in uveal melanoma is loss of chromosome 3 and BAP1: loss of chromosome 3/loss of BAP1 expression correlates with upregulation of the NFkB pathway and affects the activity of the NFkB pathway in UM tumors, which leads to upregulation of HLA Class I expression and attraction of infiltrating cells to the tumor environment which is a well known factor in the development of metastasis in this disease.

## 4. Materials and Methods

### 4.1. Study Population

Tumor tissues were obtained from 64 enucleated eyes that underwent an enucleation for UM between 1999 and 2008 at the Leiden University Medical Center (LUMC) in Leiden, The Netherlands. In our cohort of 64 patients, 51% were male and 49% were female (see Table 6 for patient and tumor characteristics of this cohort). The mean age at the time of enucleation was 61 years. The mean follow-up time (defined as the time period between enucleation and death) was 77 months (range 2 to 196 months). At the end of follow-up in 2018, 58% of patients had died because of UM metastasis, 10% because of other causes, 5% due to an unknown cause and 28% were still alive.

The collection of materials and the research protocol is compliant with the tenets of the Declaration of Helsinki (World Medical Association of Declaration 2013; ethical principles for medical research involving human subjects). Tumor material was handled in accordance with the Dutch National Ethical Guidelines (‘Code for Proper Secondary Use of Human Tissue’).

The Medisch Ethische Toetsingscommissie (METC) declared that it did not object to performing this study (19/10/2016, code G16.076/NV/gk).

### 4.2. Immunohistochemistry and Immunofluorescence

Immunohistochemical staining was previously performed for HLA-A, HLA-B and BAP1. Mouse monoclonal antibodies HCA2 (staining HLA-A heavy chains) and HC10 (staining HLA-B/C) (produced by the Netherlands Cancer institute, Amsterdam, The Netherlands), were used [36]. Tumors were divided into BAP1-positive or -negative based on nuclear staining [47]. An example of positive and negative staining is shown in van Essen et al. [48]. Briefly, tissues were incubated with mouse monoclonal antibody against human BAP1 (clone sc-28383, 1:50 dilution, Santa Cruz Biotechnology, Dallas, TX, USA). Tumor cells positive for each marker were counted at 100X magnification and were shown as a percentage of the total number of the tumor cells.

Immunofluorescence staining was performed for T cell and macrophage markers as described [9,38] with anti-CD3 (ab828; Abcam, Cambridge, MA, USA), anti-CD8 (4B11, IgG2b; Novocastra Valkenswaard, The Netherlands), anti-CD68 (514H12; Abcam, Cambridge, UK) and anti-CD163 (Clone 10D6; Novocastra, Newcastle-upon-Tyne, UK). Counts were expressed as pixels per millimeter^2^. Monoclonal antibodies were selected as described previously, based on correlations between mRNA expression and immunofluorescence staining [10].

### 4.3. Chromosome Analysis

DNA from samples collected at the Leiden University Medical Center was purified using QIAmp DNA Mini kit (Qiagen, Venlo, The Netherlands). Affymetrix 250K Nsp array (Affymetrix, Santa Clara, CA, USA) was performed in order to obtain a genome-wide micro-array of single nucleotide polymorphisms (SNPs) as described previously for chromosome 3 abnormalities [35].

### 4.4. Gene Expression

Gene expression profiling was performed with the Illumina HT12v4 array (Illumina, Inc., San Diego, CA, USA) for NFkB pathway markers and regulators of the pathway (NFkB1, NFkB2, RELA, RELB, SPP1, PPARγ, IKBKG), HLA genes and its regulators (HLA-A, HLA-B, CIITA, NLRC5, IRF1, IRF2, IRF8), and peptide-loading machinery molecules (TAP1, TAP2, Tapasin and Calreticulin), as described previously [35]. Illumina probe numbers are indicated in Supplementary Table S3.

### 4.5. Statistical Analysis

Data were analysed with SPSS software version 22.0 (SPSS, nc., Chicago, Il, USA). Graphs were obtained using GraphPad Prism version 5.0 for windows (GraphPad Software, La Jolla, CA, USA). One-Way ANOVA was used to calculate the means. Spearman correlation was performed in order to make correlations between data. Bonferroni correction was applied for multiple testing and after correction, all *p* values above one were rounded to one. The Mann–Whitney U test was used to compare non-normal groups. Kaplan–Meier survival curves were made and the log rank test was used for the significance analysis.

## 5. Conclusions

This study evaluated the association between HLA Class I expression, essential components of the NFkB signalling pathway and genetic evolution in UM. Loss of chromosome 3/loss of nuclear BAP1 protein in UM is associated with upregulation of the main components of the NFkB pathway (NFkB1-NFkB2 and RELB) and downregulation of two negative regulators of this pathway (SPP1 and PPARγ). It seems that under normal conditions, BAP1 helps to keep the uveal pigment cells immunologically quiet, but that during evolution of a UM, loss of BAP1 expression results in lack of suppression of the NFkB pathway and subsequent inflammation. Functional studies will show how BAP1 molecularly targets the NFkB pathway.

## Figures and Tables

**Table 1:** Correlation between mRNA expression levels of different NFkB pathway molecules with mRNA expression markers of HLA-A and HLA-B. *r* = two-tailed Spearman correlation coefficient. *p* ≤ 0.05 is considered significant. Bonferroni correction was applied.

Markers	HLA-A Probe 1		HLA-A Probe 2		HLA-A Probe 3		HLA-B	
	*r*	*p*	*r*	*p*	*r*	*p*	*r*	*p*
**NFkB1**	0.438	0.001	0.454	0.001	0.358	0.04	0.433	0.001
**NFkB2, pr1**	0.417	0.01	0.403	0.01	0.258	0.40	0.395	0.01
**NFkB2, pr2**	0.239	0.57	0.285	0.22	0.171	1	0.254	0.43
**RELA**	−0.055	1	−0.270	0.31	−0.280	0.25	−0.147	1
**RELB**	0.343	0.05	0.327	0.08	0.429	0.001	0.419	0.01
**SPP1, pr1**	−0.285	0.23	−0.303	0.15	−0.241	0.55	−0.325	0.09
**SPP1, pr2**	−0.311	0.12	−0.335	0.07	−0.263	0.36	−0.346	0.05
**PPARγ, pr1**	−0.325	0.09	−0.355	0.04	−0.363	0.03	−0.327	0.08
**PPARγ, pr2**	−0.294	0.18	−0.338	0.06	−0.327	0.08	−0.265	0.35
**IkBkG**	0.188	1	0.039	1	0.250	0.47	0.235	0.62

**Table 2:** Correlation between mRNA expression of NFkB pathway molecules and the antigen-presenting machinery and known regulators of HLA Class I expression, in a cohort of 64 primary UM. *r* = two-tailed Spearman correlation coefficient. *p* ≤ 0.05 is considered significant. Bonferroni correction was applied.

Markers	CIITA		NLRC5		IRF1		IRF2		IRF2, pr2		IRF8		TAP1		TAP2		Tapasin		Calreticulin	
	*r*	*p*	*r*	*p*	*r*	*p*	*r*	*p*	*r*	*p*	*r*	*p*	*r*	*p*	*r*	*p*	*r*	*p*	*r*	*p*
**NFkB1**	0.212	0.93	0.091	1	0.346	0.05	0.123	1	0.248	0.48	0.236	0.61	0.396	0.01	0.086	1	0.240	0.56	0.342	0.06
**NFkB2, pr1**	0.261	0.37	0.205	1	0.327	0.08	0.143	1	0.081	1	0.242	0.54	0.409	0.01	0.052	1	0.208	0.99	0.393	0.01
**NFkB2, pr2**	0.290	0.20	0.191	1	0.238	0.59	0.215	0.89	0.194	1	0.219	0.82	0.320	0.10	0.055	1	0.326	0.09	0.344	0.05
**RELA**	−0.249	0.48	−0.122	1	−0.180	1	0.145	1	−0.010	1	−0.175	1	−0.206	1	0.118	1	−0.011	1	0.281	0.24
**RELB**	0.106	1	0.262	0.37	0.449	0.001	0.122	1	0.082	1	0.439	0.001	0.440	0.001	0.444	0.001	0.267	0.33	0.134	1
**SPP1, pr1**	−0.255	0.42	−0.175	1	−0.394	0.01	−0.119	1	−0.081	1	−0.305	0.14	−0.439	0.001	−0.258	0.40	−0.102	1	−0.081	1
**SPP1, pr2**	−0.252	0.45	−0.174	1	−0.406	0.01	−0.134	1	−0.067	1	−0.294	0.18	−0.462	0.001	−0.260	0.38	−0.107	1	−0.089	1
**PPARγ, pr1**	−0.172	1	−0.229	0.68	−0.372	0.02	−0.062	1	0.058	1	−0.394	0.01	−0.443	0.001	−0.299	0.17	−0.259	0.39	0.147	1
**PPARγ, pr2**	−0.152	1	−0.218	0.83	−0.346	0.05	−0.082	1	−0.020	1	−0.345	0.05	−0.384	0.02	−0.263	0.36	−0.215	0.88	0.138	1
**IkBkG**	0.022	1	0.215	0.87	0.167	1	0.035	1	0.266	0.34	0.279	0.25	0.084	1	0.298	0.17	0.209	0.98	0.067	1

**Table 3:** Correlation between mRNA expression levels of various NFkB pathway molecules with mRNA expression markers of different types of infiltrating immune cells. *r* = two-tailed Spearman correlation coefficient. *p* ≤ 0.05 is considered significant. Bonferroni correction was applied.

Markers	CD3E		CD3D1		CD4		CD8A		CD68		CD163	
	*r*	*p*	*r*	*p*	*r*	*p*	*r*	*p*	*r*	*p*	*r*	*p*
**NFkB1**	0.398	0.01	0.298	0.17	0.227	0.71	0.365	0.03	0.172	1	0.009	1
**NFkB2, pr1**	0.378	0.02	0.213	0.91	0.195	1	0.258	0.39	0.375	0.02	−0.086	1
**NFkB2, pr2**	0.361	0.03	0.242	0.54	0.278	0.26	0.267	0.33	0.411	0.01	0.040	1
**RELA**	0.110	1	−0.095	1	−0.119	1	−0.125	1	−0.062	1	−0.065	1
**RELB**	0.363	0.03	0.365	0.03	0.308	0.13	0.327	0.08	0.149	1	0.317	0.11
**SPP1, pr1**	−0.295	0.18	−0.247	0.49	−0.337	0.06	−0.280	0.25	−0.442	0.001	−0.265	0.35
**SPP1, pr2**	−0.297	0.17	−0.251	0.46	−0.330	0.08	−0.281	0.25	−0.447	0.001	−0.243	0.53
**PPARγ, pr1**	−0.174	1	−0.327	0.08	−0.475	0.001	−0.256	0.41	−0.221	0.79	−0.399	0.01
**PPARγ, pr2**	−0.195	1	−0.311	0.12	−0.460	0.001	−0.276	0.27	−0.209	0.97	−0.353	0.04
**IkBkG**	0.184	1	0.196	1	0.233	0.64	0.188	1	−0.009	1	0.354	0.04

**Table 4:** Correlation between the NFkB pathway molecules and HLA Class I expression, after exclusion of one-third of the tumors with the highest infiltrate, based on CD8 expression (*n* = 43). *r* = two-tailed Spearman correlation coefficient. *p* ≤ 0.05 is considered significant.

Markers	HLA-A Probe 1		HLA-A Probe 2		HLA-A Probe 3		HLA-B	
	*r*	*p*	*r*	*p*	*r*	*p*	*r*	*p*
**NFkB1**	0.292	0.06	0.255	0.10	0.139	0.37	0.269	0.08
**NFkB2, pr1**	0.408	0.007	0.431	0.004	0.251	0.11	0.463	0.002
**NFkB2, pr2**	0.212	0.17	0.257	0.10	0.113	0.47	0.281	0.07
**RELA**	0.095	0.55	−0.067	0.67	−0.153	0.33	−0.012	0.94
**RELB**	0.097	0.54	0.188	0.23	0.199	0.20	0.248	0.11
**SPP1, pr1**	−0.238	0.12	−0.372	0.01	−0.196	0.21	−0.286	0.06
**SPP1, pr2**	−0.289	0.06	−0.425	0.01	−0.245	0.11	−0.331	0.03
**PPARγ, pr1**	−0.354	0.02	−0.431	0.004	−0.445	0.003	−0.320	0.04
**PPARγ, pr2**	−0.341	0.03	−0.403	0.007	−0.421	0.005	−0.228	0.14
**IkBkG**	0.001	1,0	−0.023	0.89	0.188	0.23	0.061	0.70

**Table 5:** Correlation between BAP1 staining as defined by immunohistochemical staining and NFkB pathway molecules expression in all tumors for which BAP1 staining was available (*n* = 55) and again after exclusion of one-third of the tumors with the highest infiltrate, based on CD8 expression (*n* = 37). One-Way ANOVA was used to calculate the means. *p* ≤ 0.05 is considered significant.

Markers	All Tumors (*n* = 55)			Exclusion of High CD8 Tumors (*n* = 37)		
	BAP1 + (Mean *n* = 25)	BAP1 − (Mean *n* = 30)	*p*	BAP1 + (Mean *n* = 22)	BAP1 − (Mean *n* = 15)	*p*
**NFKB1**	9.19	9.42	0.018	9.16	9.26	0.37
**NFKB2-pr1**	6.98	7.19	0.001	6.98	7.16	0.014
**NFKB2-pr2**	6.94	7.14	0.003	6.94	7.15	0.01
**RELA**	7.13	7.05	0.12	7.14	7.09	0.47
**RELB**	6.68	6.73	0.23	6.66	6.70	0.48
**SPP1-pr1**	10.97	8.56	<0.001	10.91	8.38	0.001
**SPP1-pr2**	10.41	7.97	<0.001	10.34	7.84	<0.001
**PPARγ-pr1**	8.15	7.25	<0.001	8.22	7.15	0.001
**PPARγ-pr2**	7.00	6.51	0.001	7.03	6.46	0.003
**IkBkG**	7.94	7.85	0.085	7.93	7.78	0.006

**Table 6:** Characteristics of the cohort of uveal melanoma (UM) patients enucleated in the LUMC between 1999 and 2008 (*n* = 64).

Characteristics		Number of Cases or Mean	% of Cases or SD
**Gender**	Male	33	51%
	Female	31	49%
**Age at enucleation (in years)**		61	±15.7
**Cell type**	Spindle	22	33%
	Mixed/epithelioid	41	62%
**Ciliary body involvement**	No	40	61%
	Yes	24	36%
**Chromosome 3 status**	Disomy	24	46%
	Monosomy	40	61%
**BAP1 Staining**	Positive	25	46%
	Negative	30	55%
	Unknown	9	16%
**GNA11 mutation**	No	31	48%
	Yes	33	52%
**GNAQ mutation**	No	36	56%
	Yes	28	44%
**Chromosome 6p gain**	No	43	65%
	Yes	21	32%
**Chromosome 8q gain**	No	19	29%
	Yes	45	68%
**Metastasis**	No	27	42%
	Yes	37	58%
**Follow-up time (in months)**		77	±55
**Vital status**	Dead due to UM metastasis	37	58%
	Dead due to other causes	6	10%
	Cause of death unknown	3	5%
	Alive at last follow-up date	18	28

## Supplementary Materials

The following are available online at https://www.mdpi.com/2072-6694/11/8/1102/s1, Table S1: Correlation between different probes obtained with an Illumina gene expression array with immunohistochemical data in the Leiden cohort (*n* = 28); Table S2: Correlation between NFkB signalling and HLA mRNA expression, after exclusion of the one-third tumors with the highest macrophage infiltrate, as indicated by CD68 mRNA levels (*n* = 43); Table S3. Illumina probe number of factors which have been used in this study.
