# Supplementary material for: Loss of BAP1 Is Associated with Upregulation of the NFkB Pathway and Increased HLA Class I Expression in Uveal Melanoma
Source: Cancers (Basel). 2019 Aug 2;11(8):1102. doi: 10.3390/cancers11081102 (PMC6721427; doi:10.3390/cancers11081102)
Supplement: Supplementary file 1 [file cancers-11-01102-s001.pdf]

Supplementary Material: Loss of BAP1 is associated with Upregulation of the NFkB Pathway and increased HLA Class I Expression in Uveal Melanoma

Zahra Souri, Annemijn P.A. Wierenga, Christiaan van Weeghel, Pieter A. van der Velden, Wilma G.M. Kroes, Gregorius P.M. Luyten, Sjoerd H. van der Burg, Aart G. Jochemsen and Martine J. Jager

Table S1. Correlation between different probes obtained with an Illumina gene expression array with immunohistochemical data in the Leiden cohort (*n* = 28) [35].

Gene-expression (Illumina)	mAb HCA2 (HLA-A)		mAb HC10 (HLA-B)	
	r	*p*	r	*p*
**HLA-A probe 1 ◊**	0.619	<0.001		
**HLA-A probe 2 ◊**	0.459	0.014		
**HLA-A probe 3**	0.055	0.781		
**HLA-A probe 4 ◊**	0.327	0.089		
**HLA-B probe ◊**			0.601	0.001

r = two- tailed Spearman correlation coefficient. $p \leq 0.05$ considered significant. ◊ selected.

Table S2. Correlation between NFkB signalling and HLA mRNA expression, after exclusion of the one-third tumors with the highest macrophage infiltrate, as indicated by CD68 mRNA levels (*n* = 43).

Markers	HLA-A Probe 1		HLA-A Probe 2		HLA-A Probe 3		HLA-B	
	r	*p*	r	*p*	r	*p*	r	*p*
**NFkB1**	0.477	**0.001**	0.372	**0.014**	0.345	**0.024**	0.446	**0.003**
**NFkB2, pr1**	0.466	**0.002**	0.393	**0.009**	0.297	0.053	0.426	**0.004**
**NFkB2, pr2**	0.159	0.309	0.140	0.371	0.072	0.648	0.178	0.253
**RELA**	−0.014	0.931	−0.150	0.339	−0.245	0.113	−0.133	0.397
**RELB**	0.112	0.473	0.182	0.242	0.259	0.093	0.207	0.182
**SPP1, pr1**	−0.393	**0.009**	−0.472	**0.001**	−0.366	**0.016**	−0.419	**0.005**
**SPP1, pr2**	−0.415	**0.006**	−0.483	**0.001**	−0.381	**0.012**	−0.429	**0.004**
**PPARγ, pr1**	−0.305	**0.046**	−0.398	**0.008**	−0.344	**0.024**	−0.268	0.082
**PPARγ, pr2**	−0.311	**0.042**	−0.386	**0.011**	−0.324	**0.034**	−0.211	0.174
**IkBkG**	0.091	0.563	0.056	0.720	0.182	0.242	0.159	0.307

r = two-tailed Spearman correlation coefficient. $p \leq 0.05$ is considered significant.

Table S3. Illumina probe number of factors which have been used in this study.

Gene-Expression (Illumina)		Probe Number
**HLA-A**	probe 1	ILMN_1671054
**HLA-A**	probe 2	ILMN_2203950
**HLA-A**	probe 3	ILMN_2186806
**HLAB**		ILMN_1778401
**CD3E**		ILMN_1739794
**CD3D**		ILMN_2261416
**CD4**		ILMN_1727284
**CD8-A**		ILMN_1768482
**CD163**		ILMN_1733270
**NFkB1**		ILMN_1714965
**NFkB2**	probe 1	ILMN_1799062

**NFkB2**	probe 2	ILMN_2390859
**RELA**		ILMN_1705266
**RELB**		ILMN_1811258
**SPP1**	probe 1	ILMN_1651354
**SPP1**	probe 2	ILMN_2374449
**PPARγ**	probe 1	ILMN_1800225
**PPARγ**	probe 2	ILMN_2364384
**IkBkG**		ILMN_1707308

© 2019 by the authors. Licensee MDPI, Basel, Switzerland. This article is an open access article distributed under the terms and conditions of the Creative Commons Attribution (CC BY) license (http://creativecommons.org/licenses/by/4.0/).
